# Development of Proteinuria in Patient With Solitary Kidney on Rosuvastatin

**DOI:** 10.1016/j.jaccas.2026.107636

**Published:** 2026-04-02

**Authors:** Joy A. Stouffer, Barinder S. Hansra

**Affiliations:** a Department of Internal Medicine, Vanderbilt University Medical Center, Nashville, Tennessee, USA; b Division of Cardiovascular Medicine, Vanderbilt University Medical Center, Nashville, Tennessee, USA

**Keywords:** acute coronary syndrome, cardiovascular disease, hypercholesterolemia

## Abstract

**Background:**

High-dose rosuvastatin (40 mg) has been associated with transient proteinuria, particularly in patients with severe chronic kidney disease.

**Case Summary:**

We report a 59-year-old woman with a solitary kidney who developed proteinuria while on rosuvastatin 40 mg daily. The reduction of the dose to 10 mg daily led to complete resolution of proteinuria.

**Discussion:**

The Food and Drug Administration recommendations advise a dose reduction of rosuvastatin to 10 mg in patients with severe chronic kidney disease, but no guidance exists for individuals with a solitary kidney and preserved renal function. This case suggests that these patients may be at risk of rosuvastatin-associated proteinuria and could benefit from lower doses.

**Take-Home Messages:**

Clinicians should use caution when prescribing high-dose rosuvastatin in patients with a solitary kidney. Further study is warranted to clarify whether solitary kidney status independently increases the risk of proteinuria.

## Visual Summary

**Figure:**
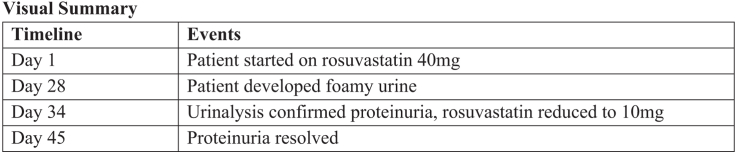

## History of Presentation

A 59-year-old woman with a history of nephrectomy in infancy (resulting in a solitary kidney) and hyperlipidemia presented with sudden-onset chest pain and diaphoresis. She was diagnosed with an inferior ST-segment elevation myocardial infarction and underwent emergent coronary angiography, receiving a drug-eluting stent to the right coronary artery and a subsequent staged intervention with a drug-eluting stent to the left anterior descending coronary artery.

**Take-Home Messages**

• Maximum-dose rosuvastatin (40 mg) can cause transient proteinuria even in patients without chronic kidney disease.
• Patients with a solitary kidney may be more susceptible to rosuvastatin-associated proteinuria despite preserved renal function.
• Further investigations are needed to establish optimal rosuvastatin dosing in patients with a solitary kidney.

She was discharged on rosuvastatin 40 mg daily, prasugrel 10 mg daily, and aspirin 81 mg daily. Renal function was near normal at the time of discharge. The serum creatinine level was 1.11 mg/dL and the estimated glomerular filtration rate (eGFR) was 57 mL/min/1.73 m^2^ on admission, improving to 0.79 mg/dL and 86 mL/min/1.73 m^2^, respectively, on discharge.

Four weeks later, the patient reported foamy urine.

## Medical History

The past medical history included nephrectomy for a nonfunctioning kidney at birth.

## Differential Diagnosis

The differential diagnosis in this case included development of chronic kidney disease manifesting as proteinuria (more likely in patients with congenital solitary kidneys), nephrotic syndrome (unlikely given lack of systemic symptoms), and a medication adverse effect. The patient's only new medications were prasugrel, aspirin, and rosuvastatin, of which only rosuvastatin is associated with proteinuria.

## Investigations

The physical examination at the 4-week follow-up appointment was unremarkable. Her basic metabolic panel (including creatine) was within normal limits. Urinalysis revealed new proteinuria (30 mg/dL) (Table 1).

**Table 1:** Urinalysis Before, During, and After Onset of Proteinuria

	February 4, 2025 (Pre-Statin)	June 27, 2025 (5 wk on Rosuvastatin 40 mg)	July 8, 2025 (2 wk After Dose Reduced to 10 mg)
Urine specific gravity	1.009	1.014	1.018
Urine pH	5.0	5.5	5.5
Urine glucose	Negative	Negative	Negative
Urine protein (mg/dL)	Negative	30	Negative
Urine ketones	Negative	Negative	Negative
Urine bilirubin	Negative	Negative	Negative
Urine leukocyte esterase	Negative	Negative	Trace
Urine nitrite	Negative	Negative	Negative
Urine blood	Negative	Trace	Negative

## Management

The rosuvastatin dose was reduced from 40 mg to 10 mg. Within 2 weeks, her foamy urine resolved, and repeat urinalysis showed complete resolution of proteinuria (Table 1).

## Discussion

High-dose rosuvastatin is known to cause transient, reversible proteinuria in a subset of patients, most of those with impaired renal function.^1^ The mechanism is incompletely understood, but inhibition of proximal tubular protein reabsorption has been proposed.^2^

Rosuvastatin is one of the most commonly prescribed medications for patients with cardiovascular disease.^3^ It is the most potent statin for lowering low-density lipoprotein cholesterol.^4^ The safety profile of rosuvastatin is similar to other statins, and it undergoes limited metabolism by the CYP450 system, leading to fewer drug-drug interactions compared with other statins.^5^ Because of these benefits, rosuvastatin is an increasingly popular choice among providers. It is common practice to dose-reduce rosuvastatin in patients with reduced renal function in order to reduce the risk of adverse events such as myopathy and rhabdomyolysis. Food and Drug Administration labeling recommends limiting rosuvastatin to 10 mg daily in patients with severe chronic kidney disease (creatinine clearance <30 mL/min/1.73 m^2^). Patients with poor renal function are at an increased risk of proteinuria with rosuvastatin initiation. In one large analysis, the incidence of proteinuria among rosuvastatin users was 1.2%, with a ninefold higher risk in patients with eGFR <30 mL/min/1.73 m^2^.^6^

Our patient had preserved renal function but a solitary kidney, which may confer increased vulnerability to nephrotoxic effects. The temporal association between high-dose rosuvastatin initiation, onset of proteinuria, and subsequent resolution after dose reduction strongly suggests causality.

This case raises the question of whether solitary kidney status should be considered when determining safe statin dosing. Although no current guidelines exist, caution with high-dose rosuvastatin may be warranted in this population.

## Conclusions

A patient with a solitary kidney but normal renal function developed proteinuria while on maximum-dose rosuvastatin, which resolved after dose reduction. Clinicians should exercise caution when prescribing high-dose rosuvastatin to patients with a solitary kidney, and further research is needed to determine whether solitary kidney status independently increases the risk of rosuvastatin-associated proteinuria.

## Funding Support and Author Disclosures

The authors have reported that they have no relationships relevant to the contents of this paper to disclose.

**Visual Summary:** Timeline of Events

Timeline	Events
Day 1	The patient started on rosuvastatin 40 mg
Day 28	She developed foamy urine
Day 34	Urinalysis confirmed proteinuria, and rosuvastatin reduced to 10 mg
Day 45	Proteinuria resolved

## References

[1] Newman C.B., Preiss D., Tobert J.A. (2019). Statin safety and associated adverse events: a scientific statement from the American Heart Association. Arterioscler Thromb Vasc Biol.

[2] Sidaway J.E., Davidson R.G., McTaggart F. (2004). Inhibitors of 3-hydroxy-3-methylglutaryl-CoA reductase reduce receptor-mediated endocytosis in opossum kidney cells. J Am Soc Nephrol.

[3] Chikatipalli R., Kuttiappan A., Kumar S. (2024). Prescribing trends and rational drug use patterns in cardiovascular patients: a cross-sectional observational study. Bioinformation.

[4] Jones P.H., Davidson M.H., Stein E.A. (2003). Comparison of the efficacy and safety of rosuvastatin versus atorvastatin, simvastatin, and pravastatin across doses (STELLAR∗ Trial). Am J Cardiol.

[5] McKenney J.M. (2005). Efficacy and safety of rosuvastatin in treatment of dyslipidemia. Am J Health Syst Pharm.

[6] Shin J.-I., Fine D.M., Sang Y. (2022). Association of rosuvastatin use with risk of hematuria and proteinuria. J Am Soc Nephrol.

